# Production of Thermophilic Chitinase by *Paenibacillus* sp. TKU052 by Bioprocessing of Chitinous Fishery Wastes and Its Application in *N*-acetyl-D-glucosamine Production

**DOI:** 10.3390/polym13183048

**Published:** 2021-09-09

**Authors:** Chien Thang Doan, Thi Ngoc Tran, San-Lang Wang

**Affiliations:** 1 Faculty of Natural Sciences and Technology, Tay Nguyen University, Buon Ma Thuot 630000, Vietnam; dcthang@ttn.edu.vn (C.T.D.); ttngoc@ttn.edu.vn (T.N.T.); 2 Department of Chemistry, Tamkang University, New Taipei City 25137, Taiwan; 3 Doctoral Program in Applied Sciences, College of Science, Tamkang University, New Taipei City 25137, Taiwan; 4 Life Science Development Center, Tamkang University, New Taipei City 25137, Taiwan

**Keywords:** chitinous fishery wastes, chitinase, crab shells, *Paenibacillus*, *N*-acetyl-D-glucosamine

## Abstract

The bioprocessing of chitinous fishery wastes (CFWs) to chitinases through fermentation approaches has gained importance owing to its great benefits in reducing the enzyme production cost, and utilizing chitin waste. In this work, our study of the chitinase production of *Paenibacillus* sp. TKU052 in the presence of different kinds of CFWs revealed a preference for demineralized crab shells powder (deCSP); furthermore, a 72 kDa chitinase was isolated from the 0.5% deCSP-containing medium. The *Paenibacillus* sp. TKU052 chitinase displayed maximum activity at 70 °C and pH 4–5, while Zn^2+^, Fe^3+^, Triton X-100, Tween 40, and SDS exerted a negative effect on its activity, whereas Mn^2+^ and 2-mercaptoethanol were found to potentially enhance the activity. Among various kinds of polysaccharide, *Paenibacillus* sp. TKU052 chitinase exhibited the best catalytic activity on colloidal chitin (CC) with K*_m_* = 9.75 mg/mL and V*_max_* = 2.43 μmol/min. The assessment of the hydrolysis of CC and *N*-acetyl chitooligosaccharides revealed that *Paenibacillus* sp. TKU052 chitinase possesses multiple catalytic functions, including exochitinase, endochitinase, and *N*-acetyl-β-D-glucosaminidase activities. Finally, the combination of *Paenibacillus* sp. TKU052 chitinase and *Streptomyces speibonae* TKU048 *N*-acetyl-β-D-glucosaminidase could efficiently convert CC to *N*-acetyl-D-glucosamine (GlcNAc) with a production yield of 94.35–98.60% in 12–24 h.

## 1. Introduction

Chitin is a linear polysaccharide made of *N*-acetyl-D-glucosamine (GlcNAc) units linked through the β-1,4-glycosidic linkages [1,2]. In nature, it is the second most abundant polysaccharide after cellulose, and is also a structural constituent of crustacean exoskeletons, squid pens, fungal cell walls, etc. [3,4]. In the fishery industry, a massive amount of chitinous fishery wastes (CFWs) are generated from shrimp shells, crab shells, and squid pens, and may cause serious environmental pollution if they are not utilized effectively [5]. The chitin extraction process from CFWs commonly involves chemical deproteinization (using strong alkalis), demineralization (using strong acids), and bleaching (using oxidants) [6], thereby potentially releasing hazardous and protein-rich wastewater. On the other hand, CFWs mainly contain chitin and proteins, which can directly serve as nutrient components for microbial fermentation [7,8,9,10]. This “green technique” has shown great potential owing to the capability of microbes in utilizing CFWs as appropriate C/N sources for producing various metabolites, such as enzymes [11,12,13,14], antioxidants [15], enzymes inhibitors [16,17], antimicrobial agents [18], etc. Concurrently, the bioremediation and chitin waste utilization aspects may be resolved through this technique [19].

GlcNAc is of interest due to its potential use in the biomedical, pharmaceutical, food, biofuel, and chemical industries [20,21]. It is commonly produced through the enzymatic and chemical hydrolysis of chitin. Compared to chemical methods requiring harsh conditions (concentrated acid and high temperature), the enzymatic methods can be operated in mild conditions, and are thus highly preferred in terms of being environmentally friendly [22]. Chitinases (EC 3.2.2.14) belong to a group of hydrolytic enzymes that catalyze the degradation of chitin by breaking down the β-1,4-glycosidic linkages between GlcNAc units [23] and have thus received much attention in the production of GlcNAc. The two categories of chitinases based on the mechanism of chitin hydrolysis include endochitinases (EC 3.2.1.14) and exochitinases (EC 3.2.1.52) [2]. While endochitinases randomly break down the β-1,4-glycosidic linkages of chitin at internal sites to release *N*-acetyl chitooligosaccharides (*N*-acetyl COSs), exochitinases—further split into two subcategories: chitobiosidases (EC 3.2.1.29) and *N*-acetyl-β-D-glucosaminidases (EC 3.2.1.30)—act at the endpoint of *N*-acetyl COSs to release (GlcNAc)_2_ (chitobiosidases) or GlcNAc (*N*-acetyl-β-D-glucosaminidases) [21]. A high yield of GlcNAc can be obtained by hydrolyzing chitin using a combination of different kinds of chitinases [22].

A wide spectrum of organisms exhibits the capacity to produce chitinase, including viruses, bacteria, insects, fungi, plants, and mammals. Among these, chitinases from microorganisms are of high industrial value, thereby attracting interest in the exploitation of their production processes [24]. For chitinase production by microbes, chitin is of considerable importance by serving as a potential carbon and nitrogen (C/N) source or an enzyme inducer [25]. Accordingly, the nutritional and enzyme-inducing functions of chitin could be excellently replaced by CFWs, which are deemed as cheap and abundantly available chitin sources. The use of CFWs for medium fermentation to produce microbial enzymes has several advantages, such as lower production costs, superior productivity, and simpler techniques [26]. Several previous studies have reported the production of microbial chitinases using different kinds of CFWs, such as shrimp shells [27,28], shrimp heads [21], crab shells [29], and squid pens [12,30,31]. *Paenibacillus,* a common chitinase-producing bacterial genus, however, has rarely been reported for chitinase production using CFWs, especially crab shells, as the unique source of C/N. As such, the evaluation of chitinase production by *Paenibacillus* using this kind of waste is of significant value.

It is of great importance to obtain novel chitinases with a low-cost production process and appropriate characteristics for industrial applications. Therefore, the objective of this study is to produce chitinase by *Paenibacillus* sp. TKU052 using CFWs, which are cheap materials, as the sole C/N source. The CFWs included in this study were shrimp head powder (SHP), demineralized shrimp shell powder (deSSP), demineralized crab shell powder (deCSP), squid pen powder (SPP), and shrimp shell powder (SSP). Furthermore, the obtained chitinase was examined for its valuable biochemical properties. The chitinase produced by *Paenibacillus* sp. TKU052 was a thermophilic enzyme with multi-functional activities, and thus its potential for saccharification of chitin was assessed. Accordingly, *Paenibacillus* sp. TKU052 chitinase was combined with *Streptomyces speibonae* TKU048 *N*-acetyl-β-D-glucosaminidase to effectively hydrolyze colloidal chitin (CC) to produce GlcNAc.

## 2. Materials and Methods

### 2.1. Materials

*Paenibacillus* sp. TKU052 was the same strain as was used in our previous work [9]. Shrimp heads were purchased from Fwu-Sow Industry (Taichung, Taiwan). Crab shells, squid pens, and shrimp shells were purchased from Shin-Ma Frozen Food Co. (I-Lan, Taiwan). The demineralization of shrimp shells and crab shells was described in our previous work [9]. Macro-Prep High S resin was purchased from Bio-Rad (Hercules, CA, USA). Chitin, 75% DDA (degree of deacetylation) chitosan, GlcNAc, 2-mercaptoethanol (2-ME), ethylenediaminetetraacetic acid (EDTA), D_2_O, Congo Red, pectin, starch, xylan, carboxymethyl cellulose, gum arabic, β-1,3-glucan, dextran and 3,5-dinitrosalicylic acid (DNS) were purchased from Sigma (St. Louis, MO, USA). Tween 20, Tween 40, Triton X-100, and SDS were purchased from Merck (Darmstadt, Germany). α-chitin powder and β-chitin powder were purchased from Charming and Beauty Co. (Taipei, Taiwan). Other chemicals used were of the highest possible quality.

### 2.2. Chitinase Assay

The activity of *Paenibacillus* sp. TKU052 chitinase was determined using CC (1%) as the substrate. Briefly, 200 µL of the reaction solution, consisting of 100 µL of CC (prepared in 100 mM sodium acetate buffer, pH = 5) and 100 µL of the enzyme, was incubated at 37 °C for 30 min. Then, 750 µL of DNS reagent was added to the reaction solution, and the mixture was heated at 100 °C for 10 min. The resulting solution was centrifuged at 13,000 rpm (10 min) to obtain a clear liquid. Then, 250 µL of the liquid was transferred to a 96-well plate and quantified by a microplate reader (Bio-Rad, Hercules, CA, USA) at 515 nm. GlcNAc was used as the reference to calculate the amount of reducing sugar in the sample solution. One chitinase unit is the amount of enzyme that catalyzes the degradation of CC to liberate 1 µM of reducing sugar at 37 °C in 1 min.

### 2.3. Chitinase Production and Purification

*Paenibacillus* sp. TKU052 was grown in a culture medium consisting of 0.05% MgSO_4_, 0.1% K_2_HPO_4_, and 1% of each CFW type (deCSP, SHP, deSSP, SSP, and SPP) or chitin at the following cultural conditions: culture temperature of 37 °C and shaking speed of 150 rpm. The culture medium was tested for its chitinase activity every 24 h. Different amounts of deCSP, from 0.25% to 3%, were also used to assess the suitable C/N concentration for chitinase production at 37 °C and 150 rpm.

One liter of *Paenibacillus* sp. TKU052 culture supernatant obtained from 5-day old culture medium was used for purifying the chitinase produced. (NH_4_)_2_SO_4_ was added to the supernatant for 60% saturation, and the mixture was kept at 4 °C overnight. The precipitate was obtained by centrifugation (4 °C, 13,000 rpm, 30 min), resuspended in 25 mM sodium phosphate buffer (pH = 5.8), dialyzed against a similar buffer, and loaded onto a Macro-Prep High S column. The elution was performed with the NaCl gradients of 0–0.1 M and 0.1–1 M. The activity fraction was concentrated by means of the freeze-drying method and loaded into a Hitachi Chromaster HPLC (High-Performance Liquid Chromatography, Hitachi, Tokyo, Japan) apparatus coupled with KW-802.5 column under the following conditions: solvent: 25 mM sodium phosphate buffer (pH = 5.8); flow rate: 0.6 mL/min; sample volume: 50 µL; column temperature: 20 °C; ultraviolet detector at the wavelength of 280 nm. The molecular weight of the *Paenibacillus* sp. TKU052 chitinase was determined according to the Laemmli method using a 10% resolving gel [9]. Zymogram of *Paenibacillus* sp. TKU052 chitinase was performed on 0.05% chitin acrylamide gel and using 0.01% Congo Red as the staining solution [30].

### 2.4. Effect of Temperature and pH

The optimum temperature for *Paenibacillus* sp. TKU052 chitinase was assayed in the range of 30–80 °C. The optimum pH for the enzyme was assayed in the range of pH = 3–10 using various buffers at the same concentration of 50 mM. The buffers included Na_2_CO_3_-NaHCO_3_ buffer (pH = 9–10), sodium phosphate buffer (pH = 6–8), sodium acetate buffer (pH = 4–5), and glycine-HCl buffer (pH = 3). The thermal and pH stabilities of *Paenibacillus* sp. TKU052 chitinase was based on the residual activity after treating the enzyme at different temperatures or pH for 1 h. The residual activity was measured at pH = 5 and 37 °C following the method described above.

### 2.5. Effect of Various Ions and Chemicals

*Paenibacillus* sp. TKU052 chitinase was incubated with various chemicals (including Fe^2+^, Ca^2+^, Ba^2+^, Mg^2+^, Cu^2+^, Fe^3+^, Zn^2+^, EDTA, 2-ME, Tween 20, Tween 40, Triton X-100, and SDS) at 20 °C for 30 min. The chemicals were tested at a final concentration of 5 mM, while the surfactants were used at 5%. The residual activity of *Paenibacillus* sp. TKU052 chitinase was measured as described above.

### 2.6. Substrate Specificity

This test was performed following the chitinase assay described above, but CC was alternately replaced by α-chitin powder, β-chitin powder, 75% DDA chitosan, 100% DDA chitosan, pectin, starch, xylan, carboxymethyl cellulose, gum arabic, β-1,3-glucan, and dextran. The K*_m_* and V*_max_* of *Paenibacillus* sp. TKU052 chitinase were estimated using Lineweaver–Burk plots with a final CC concentration range of 0.5–2.5 mg/mL.

### 2.7. The Pattern of Hydrolysis 

CC and *N*-acetyl COSs with the degrees of polymerization (DP) = 2–6 were used as the enzyme substrates to explore the hydrolysis mechanism of *Paenibacillus* sp. TKU052 chitinase. At different time intervals, the reaction solution was analyzed by the HPLC process described in Section 2.9.

### 2.8. Production of GlcNAc 

The reaction solution (20 mL), consisting of 1% CC, 100 mM sodium acetate buffer (pH 5), and 10 U/mL of *Paenibacillus* sp. TKU052 chitinase or 10 U/mL of *S. speibonae* TKU048 *N*-acetyl-β-D-glucosaminidase or 5 U/mL of *Paenibacillus* sp. TKU052 chitinase combined with 5 U/mL of *S. speibonae* TKU048 *N*-acetyl-β-D-glucosaminidase were incubated at 60 °C and a shaking speed of 150 rpm. At different time intervals, 0.2 mL of the solution was withdrawn to quantify the concentration of GlcNAc and *N*-acetyl COSs by the HPLC method (described in Section 2.9). The GlcNAc yield was calculated using the formula:

GlcNAc yield = Amount of GlcNAc/Amount of substrate (%)(1)

### 2.9. HPLC Analysis

GlcNAc and *N*-acetyl COSs were analyzed by HPLC using a Hitachi Chromaster HPLC apparatus coupled with a KS-802 column under the following conditions: solvent: water; flow rate: 0.6 mL/min; sample volume: 20 µL; column temperature: 80 °C; ultraviolet detector at the wavelength of 205 nm.

### 2.10. Proton Nuclear Magnetic Resonance (^1^H-NMR) Analysis

^1^H-NMR analysis was performed using a Bruker 600 Ultrashield NMR spectrophotometer (Bruker, New Taipei City, Taiwan). The solvent used was D_2_O and the chemical shifts are shown in ppm (parts per million).

## 3. Results and Discussion

### 3.1. Chitinase Production

**Figure 1:**
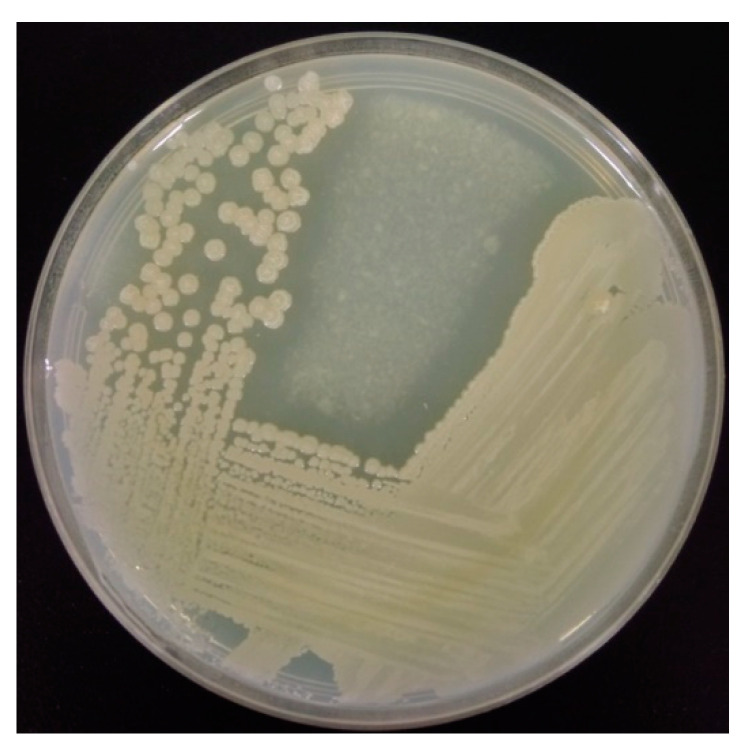
The chitinase-producing ability of *Paenibacillus* sp. TKU052 on CC agar medium.

*Paenibacillus* sp. TKU052 was isolated from the soil of Tamkang University using SPP-containing medium and was found to be closely related to *P. tyrfis* MSt1 (99.3%) and *P. elgii* SD17 (99.3%) according to 16S rDNA partial base sequence analysis [9]. In the current work, *Paenibacillus* sp. TKU052 created a zone of hydrolysis on the CC plate after three days of incubation (Figure 1), indicating that this strain secretes extracellular chitinolytic enzymes to degrade the surrounding CC. The chitinase-producing ability of *P. elgii* and *P. tyrfis* species is rarely reported [32,33]. More so, none of these species have been evaluated for their chitinase production on a medium containing CFWs and the application of their enzymes in GlcNAc production. As such, this work may provide an insight into the conversion of CFWs to produce chitinase by *Paenibacillus* sp. TKU052 strain, as well as the biochemical properties and the application of the obtained chitinase in GlcNAc production.

**Figure 2:**
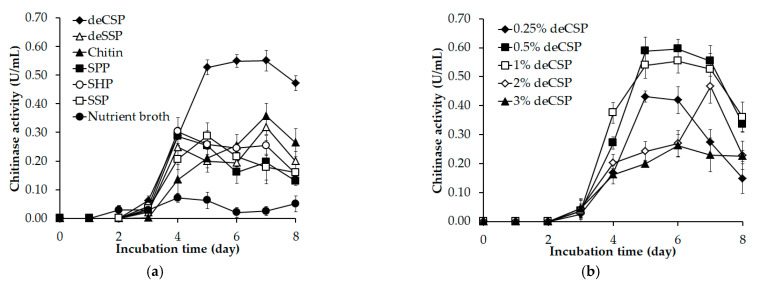
Screening of suitable C/N source for chitinase production by *Paenibacillus* sp. TKU052: (**a**) different kinds of CFWs and (**b**) different amounts of deCSP were examined. The error bar is the standard deviation of three replicates.

The chitinase production of *Paenibacillus* sp TKU052 on a medium containing CFWs as the unique C/N source was explored. The chitinous materials used were deCSP, deSSP, SPP, SHP, SSP, and chitin. In addition, nutrient broth, a non-chitinous medium, was also used to test the enzyme productivity of *Paenibacillus* sp TKU052. As shown in Figure 2a, the maximal chitinase activity of deCSP-supplemented medium was 0.53 (day 5), 0.55 (day 6), and 0.55 U/mL (day 7), that of deSSP-supplemented medium was 0.32 U/mL (day 7); that of SPP-supplemented medium was 0.29 U/mL (day 4); that of SHP-supplemented medium was 0.30 U/mL (day 4); that of SSP-supplemented medium was 0.29 U/mL (day 5); that of chitin-supplemented medium was 0.36 U/mL (day 7); and finally, that of nutrient broth medium was 0.07 (day 4) and 0.06 U/mL (day 5). According to the result, *Paenibacillus* sp. TKU052 exhibited higher chitinase productivity toward chitin-containing media, suggesting that chitin may be a key factor for chitinase production by *Paenibacillus* sp. TKU052. Among those, the highest chitinase productivity of *Paenibacillus* sp. TKU052 was found in the deCSP-supplemented medium. Chitin, especially in colloidal form, is a widely used supplement to the chitinase-producing medium as an inducer or a nutrient source. Chemical treatment is a mandatory step to produce chitin from chitinous materials, thereby possibly leading to environmental pollution and increased production cost of the processes using chitin [34,35]. Therefore, CFWs are being assessed as an effective alternative for the cost-effective production of microbial chitinases. In addition, the use of chitinous wastes for the production of microbial chitinases by various bioprocesses is regarded as being a significant part of the utilization of these waste sources. In this study, deCSP exhibited good results and was chosen as the best C/N source for chitinase production by *Paenibacillus* sp. TKU052 and further analyses.

The effect of the deCSP amount on chitinase productivity of *Paenibacillus* sp. TKU052 was examined over a range of 0.25–3%, and the results are shown in Figure 2b. The maximal chitinase activity of 0.25% deCSP-supplemented medium was 0.43 U/mL (day 5); of 0.5% deCSP-supplemented medium was 0.59 U/mL (day 5); that of 1% deCSP-supplemented medium was 0.54 U/mL (day 5); that of 2% deCSP-supplemented medium was 0.47 U/mL (day 7); and that of 3% deCSP-supplemented medium was 0.26 U/mL (day 6). The results indicate that 0.5% deCSP is the most appropriate C/N amount for chitinase production by *Paenibacillus* sp. TKU052.

With its abundance, wasted crab shell is of interest for the preparation of various bioactive compounds, such as chitin, protease, prodigiosin, antioxidant, antidiabetic and anticancer agents [9,36]. In this study, this kind of CFW was also found to be the best C/N source for chitinase production by *Paenibacillus* sp. TKU052.

### 3.2. Chitinase Purification

**Figure 3:**
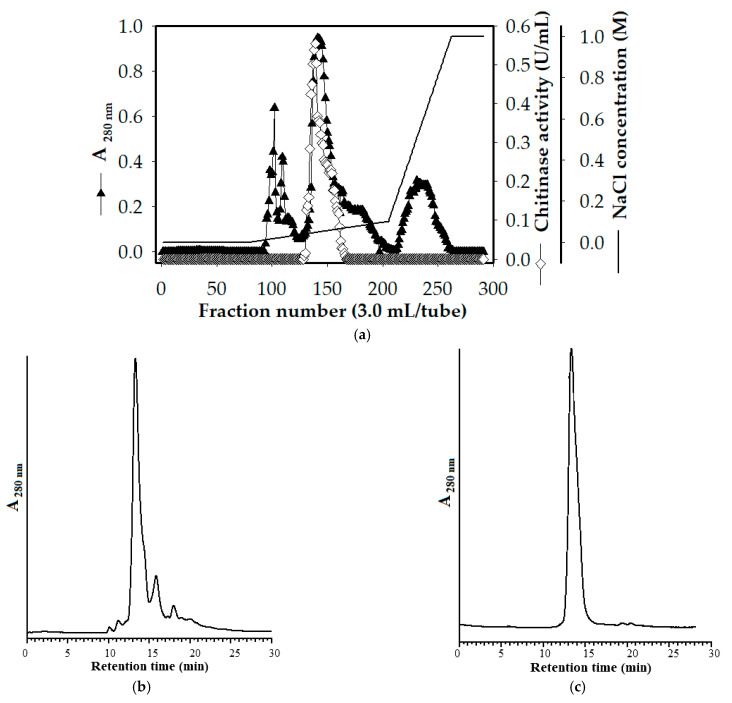
A typical cation exchange chromatography profile of the crude enzyme obtained from the supernatant of *Paenibacillus* sp. TKU052 culture medium supplemented with 0.5% deCSP (**a**); HPLC profiles of chitinase fraction obtained from the cation exchange chromatography step (**b**) and the HPLC step (**c**).

The supernatant of a 5-day old culture of *Paenibacillus* sp. TKU052 grown using 0.5% deCSP-supplemented medium was obtained by centrifugation. Chitinase in the supernatant was purified as follows: 60% (NH_4_)_2_SO_4_ precipitation, cation exchange chromatography using Macro-Prep High S resin, and size exclusion chromatography using HPLC coupling with KW-802.5 column. The crude enzyme was loaded onto a Macro-Prep High S column equilibrated by sodium phosphate buffer (25 mM, pH = 5.8). According to the enzyme elution profile, only one chitinase peak was eluted at 10–70 mM NaCl (fractions 129–166) (Figure 3a). The HPLC profile of the chitinase fraction obtained from cation exchange chromatography is shown in Figure 3b. The fraction containing the chitinase activity was eluted at a retention time of 13.3 min. The fractions that exhibited the chitinase activity were pooled and analyzed by the HPLC system using a KW-802.5 column. Accordingly, only one protein peak at a retention time of 13.3 min was observed, suggesting that the obtained chitinase was homogeneous (Figure 3c). The result of the purification of *Paenibacillus* sp. TKU052 chitinase is summarized in Table 1. Enzyme purity was estimated to be 19.47-fold greater than that of the cultural supernatant. The purified chitinase had a specific activity of 2.55 U/mg, with a yield of about 4.01%.

**Figure 4:**
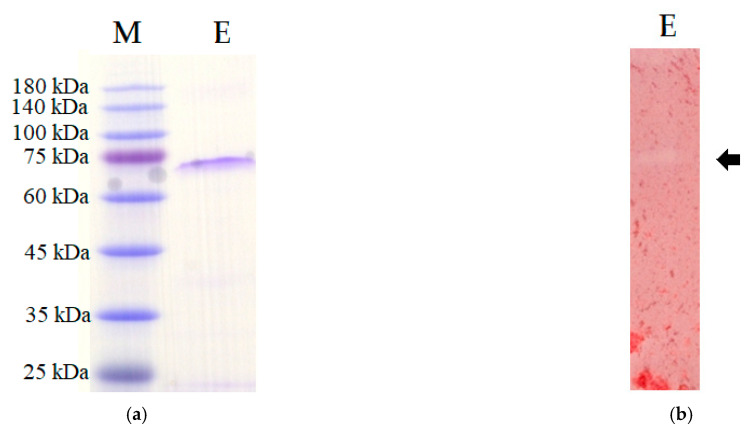
SDS-PAGE (**a**) and chitin zymography (**b**) profile of *Paenibacillus* sp. TKU052 chitinase. M, protein markers; E, the purified enzyme. The arrow indicates the enzyme location.

The homogeneity of the purified chitinase was also examined by SDS-PAGE and chitin zymography. A unique protein band (Figure 4a) and chitinolytic band (Figure 4b) at 72 kDa were observed for the purified chitinase indicating that the *Paenibacillus* sp. TKU052 chitinase has a molecule weight (MW) of 72 kDa. The MW of *Paenibacillus* sp. TKU052 chitinase is similar to that of chitinases/chitosanases from *P. barengoltzii* (74.6 kDa) [2], *P. ehimensis* MA2012 (>100, 100, 72, 65, 60, 50, 37, and 35 kDa) [37], *P. timonensis* LK-DZ15 (70 kDa) [38], *P. pasadennesis* CS0611 (69 kDa) [39], *P. elgii* HOA73 (68 kDa) [33], *Paenibacillus* sp. TKU042 (70 kDa) [35], but much higher than that from *Paenibacillus* sp. TKU047 (23 kDa) [30], *P. thermoaerophilus* TC22-2b (48 kDa) [40], *P. dendritiformis* (31 kDa) [41], *P. macerans* TKU029 (63 kDa) [42], and *P. pasadenensis* NCIM 5434 (35 kDa) [43]. Generally, the MW of chitinases/chitosanases from *Paenibacillus* species is in the range of 30–70 kDa [30].

### 3.3. Effect of Temperature and pH

**Figure 5:**
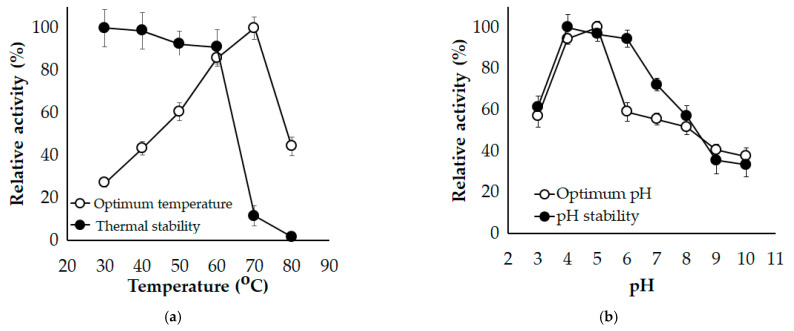
Effect of temperature (**a**) and pH (**b**) on the activity of *Paenibacillus* sp. TKU052 chitinase. The error bar is the standard deviation of three replicates.

The temperature was applied in the range of 30–80 °C to explore the influence of temperature on *Paenibacillus* sp. TKU052 chitinase activity. The optimal temperature of *Paenibacillus* sp. TKU052 chitinase was found to be 70 °C and it was stable up to 60 °C (Figure 5a). Due to the outstanding functioning at temperatures ≥ 50 °C, *Paenibacillus* sp. TKU052 chitinase could be categorized as a thermophilic enzyme. By far, thermophilic chitinolytic enzymes have gained the most attention as they can tolerate and act at elevated temperatures. The reaction at a high temperature can provide the benefits of enhancing the solubility/dispersibility of compounds and preventing microbial contamination [44]. The thermophilic enzymes have been examined for various aspects, including the source of the enzyme, the hydrolysis mechanism of the enzyme at a high temperature, genetic engineering, and industrial applications. Accordingly, several *Paenibacillus* species strains have been found to be capable of producing thermophilic chitinolytic enzymes, with promising potential in the production of chitooligosaccharides and GlcNAc, such as *P. barengoltzii* chitinase [2], *P. thermoaerphilus* TC22-2b chitinase [40], *P. mucilaginosus* TKU032 chitosanase [45], and *Paenibacillus* sp. 1794 chitosanase [46]. In this study, *Paenibacillus* sp. TKU052 chitinase exhibited a comparable or even better thermal stability and thermal activity than most of *Paenibacillus* chitinases/chitosanases [1,33,40,46,47], indicating that this enzyme may be useful for applications in which a thermophilic chitinase is prioritized.

The influence of pH on the activity of *Paenibacillus* sp. TKU052 chitinase is shown in Figure 5b. The maximal activity of the enzyme was observed within the pH range of 4–5, and it was stable within the pH range of 4–6, suggesting that *Paenibacillus* sp. TKU052 chitinase is an acidic chitinase. Likewise, several chitinolytic enzymes from *Paenibacillus* species are functional in acidic conditions, such as *P. thermoaerophilus* TC22-2b. (pH = 4) [40], *P. mucilaginosus* TKU032 (pH = 6) [45], *Paenibacillus* sp. 1794 (pH = 4.8) [46], *P. illinoisensis* KJA-424 (pH = 5) [48], *P. timonensis* LK-DZ15 (pH = 4.5) [38], *P. pasadenensis* CS0611 (pH = 5) [39], *P. barengoltzii* CAU904 (pH = 3.5) [49], *Paenibacillus* sp. D1 (pH = 5) [50], and *Paenibacillus* sp. BISR-047 (pH = 5) [51]. However, some chitinolytic enzymes from *Paenibacillus* species have their optimum pH at neutral and alkaline conditions [30,33,41,42]. Due to its pH stability and acidic pH optimum, *Paenibacillus* sp. TKU052 chitinase could be useful for the applications set up in acidic conditions.

### 3.4. Effect of Various Ions and Chemicals

The activity of *Paenibacillus* sp. TKU052 chitinase incubated with various chemicals was examined, and the results are shown in Table 2. Among the metal ions, Fe^2+^, Ca^2+^, Ba^2+^, Mg^2+^, Cu^2+^ did not significantly affect the activity of *Paenibacillus* sp. TKU052 chitinase, while Fe^3+^ and Zn^2+^ showed an inhibitory effect by reducing the enzyme activity to 26.72% and 59.51%, respectively, of its initial activity. The negative effect of heavy metal ions may be due to their ability to destroy the tertiary structure of the protein, thereby inactivating the enzyme [1]. In contrast, Mn^2+^ showed an enhancing effect on the activity of *Paenibacillus* sp. TKU052 chitinase (relative activity of 142.57%), similar to its positive effect on *P. chitinolyticus* UMBR 0002 chitinase [1] and *Paenibacillus* sp. TKU047 chitosanase [30]. Surfactants such as SDS, Triton X-100, and Tween 40 showed a negative effect on *Paenibacillus* sp. TKU052 chitinase (relative activity of 28.84%, 78.96%, and 65.71%, respectively), whereas Tween 20 did not have a significant effect. *Paenibacillus* sp. TKU052 chitinase was not significantly affected by EDTA, a metal ion chelator, indicating that the activity of the enzyme is not dependent on metal ions. Interestingly, in the presence of 2-ME, the activity of *Paenibacillus* sp. TKU052 chitinase significantly increased to 129.70% of its initial activity, demonstrating that cysteine residues are not involved in the formation of its catalytic center [52]. Earlier studies have reported strong inhibition of several chitinases by 2-ME, such as ChiA-Pt70 from *P. timonensis* LK-DZ15 [38] and CHI from *P. chitinolyticus* UMBR 0002 [1].

### 3.5. Substrate Specificity

*Paenibacillus* sp. TKU052 chitinase exhibited the highest relative activity towards CC (100.00%), followed by β-chitin powder (16.20%), 75% DDA chitosan (8.64%), and α-chitin powder (6.65%) and no activity towards other polysaccharide substrates (Table 3). This result indicates that the activity of *Paenibacillus* sp. TKU052 chitinase is specific to the linkages of GlcNAc-GlcNAc. Likewise, a similar phenomenon was observed earlier in chitinases from *P. chitinolyticus* UMBR 0002 [1], *P. barengoltzii* CAU904 [49], and *P. elgii* HOA73 [33]. In addition, the physical form of chitin significantly affected the activity of *Paenibacillus* sp. TKU052 chitinase, wherein the colloidal form of chitin was the most suitable substrate, followed by β-chitin powder and α-chitin powder. Most *Paenibacillus* chitinases exhibit the highest chitinolytic activity on CC; however, PeChi68, a chitinase from *P. elgii* HOA73, was reported to prefer chitin powder over CC [33]. Finally, the kinetic parameters (K*_m_* and V*_max_*) of *Paenibacillus* sp. TKU052 chitinase toward CC was estimated to be 9.75 mg/mL and 2.43 μmol/min, respectively.

### 3.6. Hydrolysis Pattern

**Figure 6:**
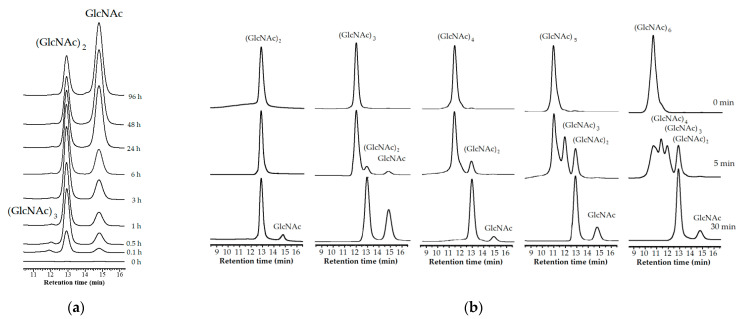
Hydrolysis pattern of *Paenibacillus* sp. TKU052 chitinase toward colloidal chitin (CC) (**a**) and *N-*acetyl COSs (**b**).

To explore the hydrolysis mechanism of *Paenibacillus* sp. TKU052 chitinase, CC and *N*-acetyl COSs (DP = 2–6) were used as the enzyme substrates. The results are shown in Figure 6. During the first stage (0–6 h), *Paenibacillus* sp. TKU052 chitinase hydrolyzed CC to yield (GlcNAc)_2_ and GlcNAc, with (GlcNAc)_2_ being the most produced product. This indicates that *Paenibacillus* sp. TKU052 chitinase is an exochitinase. From 24 h onwards, the (GlcNAc)_2_ gradually decreased over time along with the accumulation of GlcNAc, indicating a clear outcome of the hydrolysis of (GlcNAc)_2_ to form GlcNAc, which means *Paenibacillus* sp. TKU052 chitinase also possesses *N*-acetyl-β-D-glucosaminidase activity. However, the hydrolysis of (GlcNAc)_2_ to form GlcNAc of *Paenibacillus* sp. TKU052 chitinase was slow and incomplete, despite the extension of the incubation time (up to 96 h), indicating a poor *N*-acetyl-β-D-glucosaminidase activity of *Paenibacillus* sp. TKU052 chitinase. In addition, a trace peak of (GlcNAc)_3_ was detected after 0.1 h of the reaction time, suggesting that *Paenibacillus* sp. TKU052 chitinase may have some endochitinase activity. The decrease in the (GlcNAc)_3_ peak also indicates that the generated (GlcNAc)_3_ was further degraded by *Paenibacillus* sp. TKU052 chitinase. The hydrolysis mechanism of *Paenibacillus* sp. TKU052 chitinase on *N*-acetyl COSs is presented in Figure 6b. *Paenibacillus* sp. TKU052 chitinase degrades (GlcNAc)_2_ slowly to form GlcNAc, indicating its poor *N*-acetyl-β-D-glucosaminidase activity, while it could rapidly degrade (GlcNAc)_3_, (GlcNAc)_4_, (GlcNAc)_5_, and (GlcNAc)_6_ to form (GlcNAc)_2_ and GlcNAc, which confirms that it is an exochitinase. In addition, *Paenibacillus* sp. TKU052 chitinase rapidly degraded (GlcNAc)_4_ to form (GlcNAc)_2_, indicating that it could cleave (GlcNAc)_4_ at the second glycosidic linkage. *Paenibacillus* sp. TKU052 chitinase could also rapidly degrade (GlcNAc)_5_ to form (GlcNAc)_3_ and (GlcNAc)_2_, and could degrade (GlcNAc)_6_ to form (GlcNAc)_4_, (GlcNAc)_3_, and (GlcNAc)_2_, indicating that it cleaved these oligomers at the second and third glycosidic linkages. These results confirm that *Paenibacillus* sp. TKU052 chitinase may have both exochitinase and endochitinase properties. The hydrolysis mechanism of *Paenibacillus* sp. TKU052 chitinase is different from chitinases produced by *P. barengoltzii* [53], *S. speibonae* TKU048 [20], and *P. chitinolyticus* UMBR 0002 [1] but similar to those from *Chitinolyticbacter meiyuanensis* SYBC-H1 (C*m*Chi1), which has been efficiently used to prepare GlcNAc from CC [5]. In conclusion, the result suggests that *Paenibacillus* sp. TKU052 chitinase may be a multi-functional chitinase, having exochitinase, endochitinase, and *N*-acetyl-β-D-glucosaminidase activities.

### 3.7. GlcNAc Production

**Figure 7:**
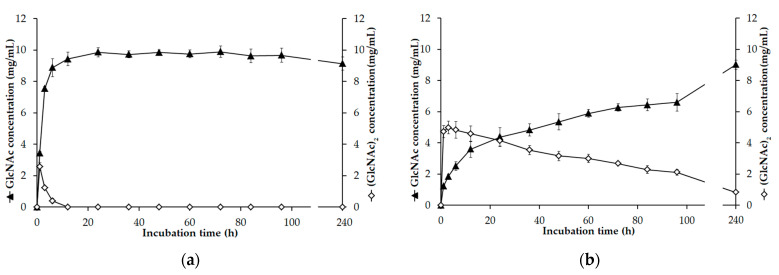

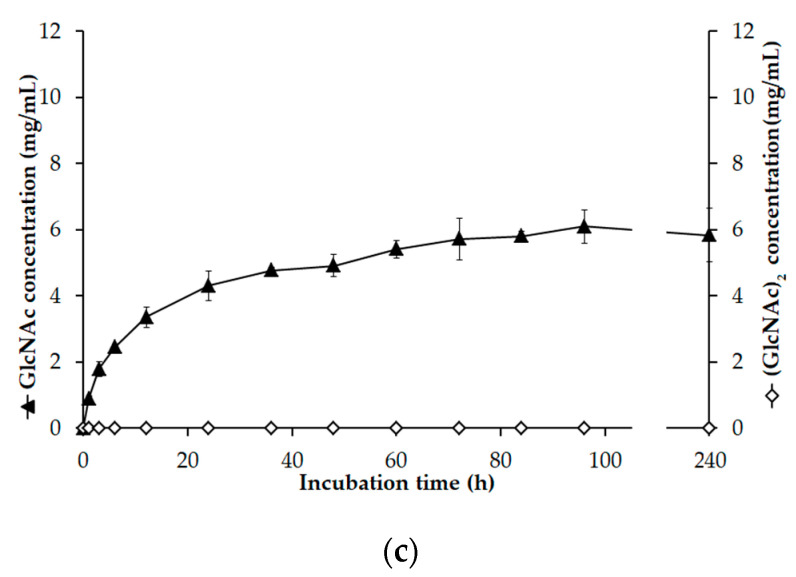
Time courses of GlcNAc and (GlcNAc)_2_ production generated from the hydrolysis of colloidal chitin (CC) catalyzed by a mixture of *Paenibacillus* sp. TKU052 chitinase and *Streptomyces speibonae* TKU048 *N*-acetyl-β-D-glucosaminidase (**a**), and independently by *Paenibacillus* sp. TKU052 chitinase (**b**), and *S. speibonae* TKU048 *N*-acetyl-β-D-glucosaminidase (**c**). The error bar indicates the standard deviation of three replicates.

**Figure 8:**
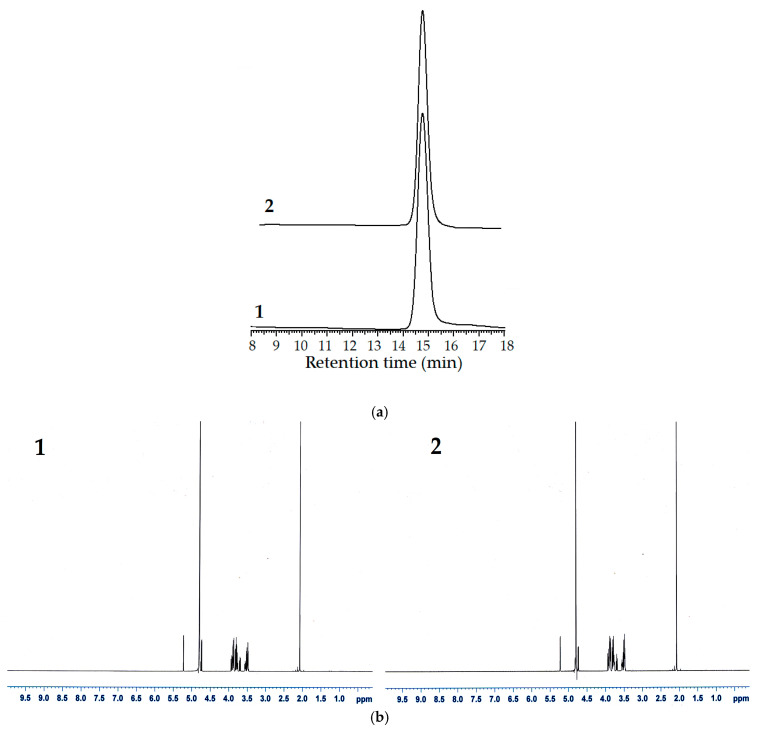
HPLC (**a**) and ^1^H-NMR (**b**) profiles of the obtained and commercial GlcNAc (1, obtained GlcNAc; 2, commercial GlcNAc).

The synergy between chitinase with both exochitinase and endochitinase activities and *N*-acetyl-β-D-glucosaminidase may be required for the complete degradation of chitin to GlcNAc [20]. According to the previous experiment, *Paenibacillus* sp. TKU052 chitinase could rapidly degrade CC to form (GlcNAc)_2_ and GlcNAc. As a result, this enzyme can effectively assist the *N*-acetyl-β-D-glucosaminidases to hydrolyze chitin and produce GlcNAc. In this study, we used a combination of *Paenibacillus* sp. TKU052 chitinase and *S. speibonae* TKU048 *N*-acetyl-β-D-glucosaminidase to assess the possibility of producing GlcNAc from CC. The products of the hydrolysis process were analyzed by the HPLC method, and the results are presented in Figure 7. In the first hour, GlcNAc and (GlcNAc)_2_ were generated at the concentrations of 3.44 and 2.58 mg/mL, respectively. In the next few hours, the concentration of (GlcNAc)_2_ significantly decreased and reduced to zero after 12 h, whereas the concentration of GlcNAc gradually increased over time and reached its maximum value after 24 h (9.86 mg/mL). According to the estimates, the GlcNAc production process could achieve a yield of 98.60% within a reaction time of 24 h. In addition, 9.44 mg/mL of GlcNAc, indicating a production yield of 94.35%, without *N*-acetyl COSs could be observed after a reaction time of 12 h. In addition, we also explored the hydrolysis processes of CC using *Paenibacillus* sp. TKU052 chitinase and *S. speibonae* TKU048 *N*-acetyl-β-D-glucosaminidase separately. *Paenibacillus* sp. TKU052 chitinase could not completely degrade (GlcNAc)_2_, and the reaction solution still contained both (GlcNAc)_2_ and GlcNAc at 0.83 and 9.03 mg/mL concentrations, respectively, (after 240 h). In the case of *S. speibonae* TKU048 *N*-acetyl-β-D-glucosaminidase, 6.10 mg/mL of GlcNAc without *N*-acetyl COSs was obtained from the CC hydrolysis with a 60.99% yield after 96 h. This indicates that a novel combination of *Paenibacillus* sp. TKU052 chitinase and *S. speibonae* TKU048 *N*-acetyl-β-D-glucosaminidase could efficiently improve the GlcNAc production from CC. The efficiency of the present process was compared with those in earlier reports (Table 4). Among them, the combination of *Paenibacillus* sp. TKU052 chitinase and *S. speibonae* TKU048 *N*-acetyl-β-D-glucosaminidase reveals a comparable result with a short required reaction time (12–24 h) and high GlcNAc production yield (94.35–98.60%). In addition, the absence of *N*-acetyl COSs in the final reaction solution could also simplify the GlcNAc purification step. In this study, GlcNAc was obtained from the chitin hydrolysate solution by one-step purification using HPLC coupled with a KS-802 column. The HPLC result revealed only one peak at a retention time of 14.7 min (Figure 8a), indicating that the molecular weight of the product is similar to that of the GlcNAc standard. The obtained GlcNAc was analyzed by ^1^H-NMR spectroscopy, and as shown in Figure 8b, the obtained GlcNAc had a similar ^1^H-NMR profile as that of the profile of commercial GlcNAc from Sigma (St. Louis, MO, USA), confirming the purity of the product. This result, therefore, provides a promising enzymatic method for the production of GlcNAc from CC.

## 4. Conclusions

The exploitation of CFWs to obtain high-quality products through microbial fermentation is increasing gradually. In this study, deCSP was found to be the best potential C/N source for chitinase production by *Paenibacillus* sp. TKU052 among several CFWs, and a chitinase with an MW of 72 kDa was purified from the 0.5% deCSP-containing medium. Interestingly, the pure chitinase exhibited some valuable properties, such as high catalytic function at acidic pH and elevated-temperature conditions (pH = 4–5, and 70 °C), and can also carry out multi-functional (exochitinase, endochitinase, and *N*-acetyl-β-D-glucosaminidase) activities. These characteristics make *Paenibacillus* sp. TKU052 chitinase a promising candidate for the saccharification of chitin under mild conditions. Remarkably, the coupling of *Paenibacillus* sp. TKU052 chitinase and *S. speibonae* TKU048 *N*-acetyl-β-D-glucosaminidase could efficiently improve the GlcNAc production from CC with a yield of 94.35–98.60% in 12–24 h. As a result, the bioprocessing of deCSP by *Paenibacillus* sp. TKU052 may be potentially useful in producing chitinase as a tool for the bioconversion of chitin to GlcNAc.

## Figures and Tables

**Table 1:** A summary of the purification of *Paenibacillus* sp. TKU052 chitinase.

Step	Total Protein (mg)	Total Activity (U)	Specific Activity (U/mg)	Recovery (%)	Purification (Fold)
Cultural supernatant	2731.95	358.00	0.13	100.00	1.00
(NH_4_)_2_SO_4_ precipitation	395.94	164.40	0.42	45.92	3.17
Macro-Prep High S	39.67	43.56	1.10	12.17	8.38
KW-802.5	5.63	14.36	2.55	4.01	19.47

**Table 2:** Effect of various chemicals on the activity of *Paenibacillus* sp. TKU052 chitinase.

Chemical	Relative Activity (%)
None	100.00 ± 7.23
Fe^2+^	102.31 ± 3.85
Fe^3+^	26.72 ± 4.12
Ca^2+^	102.13 ± 6.37
Ba^2+^	107.57 ± 3.12
Mn^2+^	142.57 ± 5.02
Mg^2+^	102.65 ± 2.18
Cu^2+^	109.93 ± 6.82
Zn^2+^	59.51 ± 4.30
SDS	28.84 ± 3.30
Triton X-100	78.96 ± 7.48
Tween 40	65.71 ± 11.06
Tween 20	101.16 ±12.15
EDTA	108.69 ± 7.21
2-ME	129.70 ± 5.45

The data are presented as mean ± standard deviation.

**Table 3:** The activity of *Paenibacillus* sp. TKU052 chitinase on different kinds of substrates.

Substrate	Relative Activity (%)
CC	100.00 ± 8.37
α-chitin powder	6.65 ± 0.48
β-chitin powder	16.20 ± 6.36
75% DDA chitosan	8.64 ± 4.46
100% DDA chitosan	N.A.
Pectin	N.A.
Starch	N.A.
Xylan	N.A.
Carboxymethyl cellulose	N.A.
Gum arabic	N.A.
β-1,3-glucan	N.A.
Dextran	N.A

N.A., no activity. The data are presented as mean ± standard deviation.

**Table 4:** Comparison of enzymatic GlcNAc production by different microbial chitinases.

Enzyme	Substrate	Time Consumed	Yield (%)	Ref.
*Paenibacillus* sp. TKU052 chitinase and *S. speibonae* TKU048 *N*-acetyl-β-D-glucosaminidase	CC	12–24 h	94.35–98.60	This study
*S. speibonae* TKU048 *N*-acetyl-β-D-glucosaminidase	β-chitin powder	96 h	73.64	[20]
*Serratia marcescens* chitinases (SmChiA, SmChiB, and SmChiC) and *Ostrinia furnacalis N*-acetyl-d-glucosaminidase (OfHex1)	*Asperillus niger* mycelia powder	24 h	93	[22]
*Streptomyces coelicolor* A3(2) chitinase C (ScChiC) and *N*-acetylhexosaminidase (ScHEX)	Crystalline chitin	8 h	90	[54]
*Chitinolyticbacter meiyuanensis* SYBC-H1 chitinase (CmChi1)	CC	24 h	98	[5]
*C. meiyuanensis* SYBC-H1 chitinase	Chitin powder	4 days	near 100	[55]
*Myceliophthora thermophila* C1 β-*N*-acetylglucosaminidase (M*th*NAG) and chitinase Chi1	Swollen chitin	24 h	37.8	[56]
*Aspergillus terreus* chitinase	Ground prawn shell	5 days	30	[57]
	Chitin flakes		73	
	Colloidal prawn shell		80	
	Swollen chitin		92	
*Aeromonas* sp. PTCC1691 crude enzyme	CC	24 h	79	[58]
*Aeromonas caviae* CHZ306 enzyme cocktail	CC	6 h	90	[59]
*Aeromonas* sp. GJ-18 crude enzyme	Swollen chitin	5–9 days	83.0–94.9	[60]
*P. barengoltzii* β*-N-*acetylglucosaminidase (PbNag39) and chitinase (PbChi70)	Powdery chitin	24 h	75.3	[61]
	CC		97.0	
*P. barengoltzii* chitinase (PbChi74) and *Rhizomucor miehei* β-*N*-acetylglucosaminidase (RmNAG)	CC	24 h	92.6	[2]
*P. illinoisensis* KJA-424 crude enzyme	Swollen chitin	24 h	62.2	[62]
*Streptomyces alfalae* β*-N-*acetylhexosaminidase (SaHEX) and a commercial chitinase (SgCtn)	CC	6 h	93.7	[63]
*Streptomyces violascens* β*-N-*acetylglucosaminidases (SvNag2557 and SvNag4755)	CC		80.2	[64]
	Ionic liquid pretreated chitin		73.8	

## Data Availability

The data presented in this study are available on request from the corresponding author.
